# Forced convection of non-darcy flow of ethylene glycol conveying copper(II) oxide and titanium dioxide nanoparticles subject to lorentz force on wedges: Non-newtonian casson model

**DOI:** 10.3389/fchem.2022.1010591

**Published:** 2022-09-26

**Authors:** Parvaiz Ahmad Naik, N. Indumathi, B. Ganga, S. Charles, A. K. Abdul Hakeem, Zahoor Iqbal, ElSayed Tag-ElDin, Jian Zu

**Affiliations:** ^1^ School of Mathematics and Statistics, Xi’an Jiaotong University, Xi’an, China; ^2^ Department of Mathematical Sri Ramakrishna Engineering College, Coimbatore, India; ^3^ Department of Mathematics, Providence College for Women, Coonoor, India; ^4^ Department of Mathematics, PSG College of Arts and Science, Coimbatore, India; ^5^ Department of Mathematics, Sri Ramakrishna Mission Vidyalaya College of Arts and Science, Coimbatore, India; ^6^ Department of Mathematics, Quaid-i-Azam University, Islamabad, Pakistan; ^7^ Faculty of Engineering and Technology, Future University in Egypt New Cairo, New Cairo, Egypt

**Keywords:** casson CuO-TiO_2_/C_2_H_6_O_2_, viscous dissipation, radiation, non-Darcy porous medium, MHD, wedge

## Abstract

The topic of two-dimensional steady laminar MHD boundary layer flow across a wedge with non-Newtonian hybrid nanoliquid (CuO-TiO_2_/C_2_H_6_O_2_) with viscous dissipation and radiation is taken into consideration. The controlling partial differential equations have been converted to non-linear higher-order ordinary differential equations using the appropriate similarity transformations. It is demonstrated that a number of thermo-physical characteristics govern the transmuted model. The issue is then mathematically resolved. When the method’s accuracy is compared to results that have already been published, an excellent agreement is found. While the thermal distribution increases with an increase in Eckert number, radiation and porosity parameters, the velocity distribution decreases as porosity increases.

## 1 Introduction

A solid-liquid dispersion of 1–100 *nm* sized nanoparticles or nanofibers makes up nanofluids. Due to the distinct physical and chemical properties of nanometer-sized particles, nanofluids have a wide range of commercial uses. Because of the improvement in their thermal properties, nanofluids have gained a lot of attention. This area has been the subject of extensive research. Fluids with low thermal conductivity include blends of water, oil, and ethylene glycol. These liquids serve as a cooling technique that boosts productivity and lowers operational expenses. Ahmadi et al. (2018) reviewed theoretically and experimentally the influential parameters on the heat conductivity of numerous nanofluids. According to their results, greater temperatures and nanoparticle concentrations typically result in nanofluids with better thermal conductivities. Philip et al. (2016) examined nitrate salts doped with CuO nanoparticles for thermal energy storage with better heat transmission problems. Shaw et al. (2022) studied boundary layer flow caused by radial stretching in TiO_2_ nanotubes-water nanofluid. They observed that the momentum transmission of that nanofluid is controlled while using the non-Newtonian fluid model. Aladdin and Bachok (2020) reported the range of dual solutions expand widely for suction and closely reduce for solid volume fraction of Al_2_O_3_ and TiO_2_ nanoparticles. Nishant and Shriram (2016) found that the total heat transfer coefficient and thermal conductivity of water and ethylene glycol-based nanofluids by the use of CuO and TiO_2_ nanoparticles are enhanced.

A hybrid nanofluid is made up of a base-fluid and two or more nanoparticles. A new class of such mixtures known as hybrid nanofluids has just been created. Compared to nanofluids, this class of materials has more effective heat transfer characteristics. Eshgarf et al. (1959) provided an excellent review of hybrid nanofluid properties, preparation, models, and stability. They found that the hybrid nanofluids had much better thermal conductivity than the traditional nanofluids (single particles). Additionally, it was shown that the choice of base fluid affects the thermal conductivity of the hybrid nanofluids. Mishra et al. (2022) observed that the Cu-Al_2_O_3_/EG hybrid nanofluid flowing through a rotating channel exhibits a considerable increase in the shear rate as well as the rate of heat transfer. Khashi’ie et al. (2022) investigated the thermal progression of a second-grade hybrid Cu-Al_2_O_3_/CH_3_OH nanofluid towards a permeable surface. It has been shown by Alhowaity et al. (2022) that the velocity and heat conduction rate through a stretching surface are greatly increased by the dispersion of copper and graphene nanoparticulates into the base fluid ethylene glycol using a power-law non-Newtonian model.

There are numerous thermal engineering applications where fluid flows across wedge-shaped bodies happen, including geothermal systems, crude oil extraction, thermal insulation, heat exchangers, and the storage of nuclear waste, etc., Holden (1970) done an experimental as well as the theoretical review on boundary layer flow on wedge, flat plate and sphere in 1970. Both the joined and detached interaction zones over the models were thoroughly examined by them. He resulted that the separation first happened on the compression surface downwind of the flat plate and that it was not always possible to determine early separation by looking for the first time that a pressure distribution inflexion point appeared. Romanov and Kolesnikova (2021) reviewed the boundary value problems past a wedge and briefly discussed on the properties and applications. Xiu et al. (2022) evaluated the forced convection flow of water-based ternary hybrid nanofluid on wedge surfaces at various temperatures with a focus on the comparative examination of large and small volume of nanoparticles. It was found that forced convection flow on wedges has higher friction linked with higher velocity as time grows long, regardless of whether the volume of nano-particles is large or tiny. Ali and Abdul Alim (2022) investigated above the wedge surface and finalised that, particularly mixed convection, thermophoresis and heat generation parameters, have no effect on skin friction. Haq et al. (2022) researched and discussed that the increment in angle of rotation enhances the local Nusselt number.

Convection is the movement of fluid that allows heat to move from one location to another. Convective heat transfer combines the processes of conduction and advection, although it is frequently mentioned as a separate type of heat transfer. Convective heat transfer plays a significant role in processes involving high temperatures. Convective boundary conditions are more useful in material drying, transpiration cooling operations and other industrial and engineering processes. In a forced convection mechanism or kind of transport, fluid motion is produced by an outside source. Many researchers have participated in the investigations into the impact of forced convection as a result of the outstanding concept. Khurana et al. (2017) reviewed on TiO_2_, CuO, Al_2_O_3_ forced convection thermal transfer and resulted that the researchers employed less than 3% volume concentration in their tests because nanofluids can improve heat transmission at low particle concentrations. Cui et al. (2022), Khan et al. (2022), Yasir et al. (2022), Ahmad et al. (2022), Hoi et al. (2022), Zhang et al. (2022) have studied the role that the effect of forced convection plays on different fluid kinds and surfaces.

Radiation is the release of energy in the form of electromagnetic waves or ionising particles, especially high-energy particles. Chamkha (2000) looked at and explored how thermal radiation and buoyancy impacted hydromagnetic flow over a permeable surface that was accelerating and had a heat source or sink. It was discovered that the wall heat transfer was reduced as a result of thermal radiation, positive wall mass transfer, magnetic field, or heat creation. In the presence of Soret and Dufour’s effects, Chamkha and Ben-Nakhi (2008) studied the MHD mixed convection-radiation interaction along a permeable surface submerged in a porous liquid. They came to the conclusion that thermal radiation decreased the local Nusselt number and increased the local Sherwood number, particularly when suction was present. Heat and mass transfer analysis of steady/unsteady hybrid nanofluid (MWCNT-Ag/water) flow across a stretching sheet with thermal radiation was covered by Sreedevi et al. (2020). They discovered that for both steady-state and unstable instances, the thermal boundary layer thickness increases with cumulating values of radiation. Numerous studies on flow and heat transfer over various geometries in the presence of radiation for mono/hybrid nanofluids have been published [see, for instance, (Chamkha et al., 2003; Al-Mdallal et al., 2020; Khan et al., 2020; Algehyne et al., 2022; Hamad et al., 2022)].

Heat and mass transmission in porous media have been the focus of several investigations. Due to their widespread occurrence in industrial and technological applications, geothermal reservoirs, drying of porous solids, thermal insulation, increased oil recovery, packed-bed catalytic reactors, and many other applications are examples of certain applications. Along with the size of the potential gradient passing through the porous medium, drag forces encountered by the fluid inside the porous medium also affect how fluid flows through it. The two basic categories of drag forces that oppose fluid movement are surface drag (friction) and drag caused by solid barriers [see Rasool et al. (2022)]. The Darcy equation simply takes into account surface drag in its simplest version. Darcy’s equation is therefore only applicable for Reynolds numbers lower than one. Solid barrier drag is comparable to surface drag at higher velocities, or for Reynolds numbers greater than one. The nonlinear term known as the Forchheimer term describes the drag pressure drop. When high velocities are present, non-Darcy behaviour is crucial for explaining fluid flow in porous media. The free and forced convection nanofluid flow across a non-Darcian porous surface, which is based on the laminar boundary-layer concept, has been the subject of extensive research. Consider, for example, Chamkhax (1996), Chamkha (1997), Chamkha et al. (2006), Modather et al. (2009), Kandasamy et al. (2014), Qawasmeh et al. (2019).

When a magnetic field is applied to a fluid flow, an electromotive force is produced, altering the distribution of velocities. A magnetic field applied in the transverse direction of the flow, as opposed to one applied in the direction of the flow, directly affects the fluid’s velocity and may be more effective at controlling the flow. However, it requires more energy and increases drag forces [see Jha and Aina (2018)]. The interaction between the electrically conducting fluid and a magnetic field has an impact on several industrial equipment types, including boundary layer control, pumps, bearings, and MHD generators. Mittal et al. (2018) completed their review of the magnetohydrodynamic flow in nanofluids. They came to the conclusion that the magnetic field’s strength assisted the concentration rise as the velocity and temperature fell due to the Lorentz forces. The effects of Hall and ion slip on unstable MHD free convective rotating flow through a saturated porous media over an exponentially accelerating plate were discussed by Veera Krishna et al. (2020). It is reported that a magnetic field is said to prevent the stream from turning around. Several well-known authors (Chamkha, 1997), (Veera Krishna et al., 2018), (Ashraf et al., 2022), (Veera Krishna and Chamkha, 2020), (Veera Krishna and Chamkha, 2019), (Veera Krishna et al., 2021) addressed the MHD flow of electrically conducting liquids in various arrangements.

The viscosity of the fluid transforms some kinetic energy into thermal energy while fluid particles are in motion. Viscous dissipation is the term used to describe this irreversible process, which is induced by viscosity (Animasaun et al. (2022)). According to Koriko et al. (2021) an increase in the Eckert number implies an increase in the rate at which kinetic energy is being converted to internal energy. This is capable of boosting temperature distribution across the dynamics of blood-gold Carreau nanofluid and dusty fluid.

Any categorised fluid can attain density increases of two or three orders of magnitude, which are not insignificant in the polymer phase. Non-Newtonian fluids do not adhere to the law of viscosity. As a result, the idea of non-Newtonian dynamics was popularised by Isaac Newton. Most applications of non-Newtonian viscosity qualities are found in the field of biochemical engineering. According to Adewale et al. (2017), who tested the models for non-Newtonian liquids, the Casson physiochemical model accurately characterised in both low and high tensile conditions. Some other relevant studies are mentioned in (Lin and Lin, 1987; Chamkha and Ben-Nakhi, 2008; Iqbal et al., 2020a; Iqbal et al., 2020b; Kumar et al., 2020; Wakif et al., 2020; Alghamdi et al., 2021; Wakif et al., 2021; Zaydan et al., 2022).

Because of the importance of the above-mentioned factors, the main contribution of this research is an investigation of the non-Newtonian viscous dissipative mono nanoliquid TiO_2_/C_2_H_6_O_2_ and hybrid nanoliquid CuO - TiO_2_/C_2_H_6_O_2_ and subjected to an MHD forced convective flow past a non-Darcian wedge with radiation effects numerically. Therefore, this study is conducted to explore that what are the impacts of the non-Newtonian TiO_2_/C2H6O_2_ and CuO - TiO_2_/C_2_H_6_O_2_ nanoliquids boundary layer flow past the wedge with radiation and viscous dissipation effects?

## 2 Mathematical formulation

**FIGURE 1:**
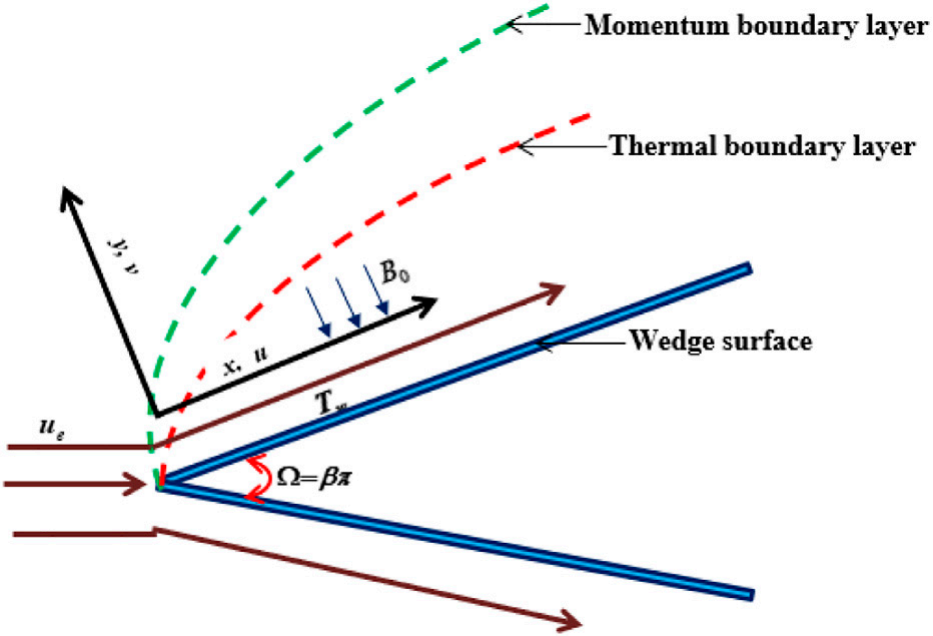
Physical Configuration.

A steady 2-D MHD boundary layer flow of a Casson ethylene glycol-based hybrid *CuO-TiO*_*2*_nanoliquid/TiO_2_nanoliquid over a wedge is studied at velocity *u* and $u_{e}=Ax^{m}$ reflects the atmospheric nanofluid velocity. The Cartesian coordinate system (*x,y*) is used, where the positive *y*-axis is taken normal to the wedge and the *x*-axis is picked along the wedge. The $T_{w}$ and $T_{\infty }$ represent the temperature of the wedge and the ambient fluid, respectively, as illustrated in Figure 1. The tensile state equation for Casson’s hybrid nanoliquid shape is as follows: [See (Xiu et al., 2022)]

$$\tau_{cd}=\left\{ \begin{array}{l} 2\left(\mu_{P}^{*}+\frac{S_{y}^{*}}{\sqrt{2\pi }}\right)e_{cd},\pi >\pi_{c}^{*}\\ 2\left(\mu_{P}^{*}+\frac{S_{y}^{*}}{\sqrt{2\pi_{c}^{*}}}\right)e_{cd},\pi >\pi_{c}^{*} \end{array}\right.$$ (1)

where $\pi_{cd}=e_{cd}e_{cd}$ is ${\left(c,d\right)}^{th}$ a component of deformation and $\pi_{c}^{*}$ is called the critical value, $S_{y}^{*}$ is the yield stress and $\mu_{P}^{*}$ is the plastic viscosity. The base liquid (Ethylene Glycol) is in thermal balance with the nanoparticles (cupric oxide *CuO* and titania *TiO*_*2*_) and there is no contact between the two solid components. Table 1 shows *CuO*, *TiO*_*2*_ and *EG* (nanoparticles and base liquid) thermophysical properties. It is assumed that the applied magnetic field $B_{0}$ is homogenous and that it is pointing in the direction normal to the surface. The maximum angle of the wedge is represented by $\Omega =\beta_{1}\pi$ , where $\beta_{1}$ is the Hartree pressure gradient. So, the set of governing equations of the hybrid nanoliquid movement are (Al-Mdallal et al., 2020), (Kandasamy et al., 2014) and (Qawasmeh et al., 2019)

$$u_{x}+v_{y}=0$$ (2)

$$\begin{array}{l} uu_{x}+vu_{y}=u_{e}\frac{du_{e}}{dx}+\frac{\mu_{hnl}}{\rho_{hnl}}\varepsilon \left(1+\frac{1}{\beta }\right)u_{yy}-\upsilon_{hnl}\frac{\varepsilon^{2}}{K}\left(1+\frac{1}{\beta }\right)\left(u-u_{e}\right)\\ -F\left(u^{2}-u_{e}^{2}\right)-\frac{\sigma_{hnl}B_{0}^{2}\left(u-u_{e}\right)}{\rho_{hnl}} \end{array}$$ (3)

$$uT_{x}+vT_{y}=\alpha_{hnl}T_{yy}+\frac{\mu_{hnl}}{{\left(\rho C_{p}\right)}_{hnl}}\left(1+\frac{1}{\beta }\right)u_{yy}-\frac{1}{{\left(\rho C_{p}\right)}_{hnl}}{\left(q_{r}\right)}_{y}$$ (4)

**TABLE 1:** Thermophysical properties of nanoparticles and base liquid Khan et al. (2022).

Properties	$\rho \left(kg/m^{2}\right)$	$C_{p}\left(J/kgK\right)$	k(W/mK)	σ(S/m)	Pr
CuO	6,510	540	18	5.96 $\times 10^{7}$	
TiO_2_	4,250	686.2	8.9538	2.38 $\times 10^{6}$	
EG	1,113.5	2,430	0.252	5.5 $\times 10^{-6}$	204

Since the frequency of electron-atom collisions is predicted to be minimal, Hall and ion slip currents can be disregarded. In dimensional patterns, the initial and boundary requirements with forced convection can be expressed as. $u=v=0,\;T=T_{w}$ at y=0

$$u\to u_{e},T\to {\;T}_{\infty }\;as\;y\to \infty$$ (5)

Here u and v are velocity components along the x and y axes, respectively. $u_{e}=Ax^{m}$ is the potential flow velocity, where $\beta_{1}=\frac{2m}{m+1}$ , m is a constant, Qawasmeh et al. (2019), β is the Casson liquid parameter, $\upsilon ,\rho ,\alpha ,\mu ,C_{P}$ and $B_{0}$ are kinematic viscosity, density, thermal diffusivity, dynamic viscosity, specific heat and magnetic field capacity respectively. The thermophysical properties of nanoliquid and hybrid nanoliquid is tabulated in Table 2. The subscript hnl denotes hybrid nanoliquid $\left(TiO_{2}-CuO/EG\right)$ and nl denotes nanoliquid. The subscripts x and y denotes the partial differentiation with respect to x and y respectively. $K,\varepsilon ,F=C_{b}\varepsilon^{2}/\sqrt{K}$ are permeability, porosity and Forchheimer coefficient of porous media respectively. Whereas, $C_{b}$ is the drag coefficient.

**TABLE 2:** Thermophysical properties of hybrid nanoliquid and nanoliquid Al-Mdallal et al. (2020).

Properties	Hybrid nanoliquid (TiO_2_-CuO/EG)
$\mu_{hnl}$	$\frac{\mu_{l}}{{\left(1-\varphi_{1}\right)}^{2.5}{\left(1-\varphi_{2}\right)}^{2.5}}$
$\rho_{hnl}$	$\left\{\left(1-\varphi_{2}\right)\left[\rho_{s_{1}}+\left(1-\varphi_{1}\right)\rho_{l}\right]\right\}+\varphi_{2}\rho_{s_{2}}$
$\rho C_{hnl}$	$\left\{\left(1-\varphi_{2}\right)\left[\varphi_{1}\rho C_{s_{1}}+\left(1-\varphi_{1}\right)\right]\rho C_{l}\right]+\varphi_{2}\rho C_{s_{2}}$
$\alpha_{hnl}$	$\frac{k_{hnl}}{{\left(\rho C_{p}\right)}_{hnl}}$
$\frac{\sigma_{hnl}}{\sigma_{nl}}$	$\frac{\sigma_{s_{2}}+2\sigma_{nl}-2\varphi_{2}\left(\sigma_{nl}-\sigma_{s_{2}}\right)}{\sigma_{s_{2}}+2\sigma_{nl}+\varphi_{2}\left(\sigma_{nl}-\sigma_{s_{2}}\right)}$
	Where $\frac{\sigma_{nl}}{\sigma_{l}}=\frac{\sigma_{s_{1}}+2\sigma_{l}-2\varphi_{1}\left(\sigma_{l}-\sigma_{s_{1}}\right)}{\sigma_{s_{1}}+2\sigma_{l}+\varphi_{1}\left(\sigma_{l}-\sigma_{s_{1}}\right)}$
$\frac{k_{hnl}}{k_{nl}}$	$\frac{k_{s_{2}}+\left(n-1\right)k_{nl}-\left(n-1\right)\varphi_{2}\left(k_{nl}-k_{s_{2}}\right)}{k_{s_{2}}+\left(n-1\right)k_{nl}+\varphi_{2}\left(k_{nl}-k_{s_{2}}\right)}$
	Where $\frac{k_{nl}}{k_{l}}=\frac{k_{s_{1}}+\left(n-1\right)k_{l}-\left(n-1\right)\varphi_{1}\left(k_{l}-k_{s_{1}}\right)}{k_{s_{1}}+\left(n-1\right)k_{l}+\varphi_{1}\left(k_{l}-k_{s_{1}}\right)}$
Properties	Nanoliquid (TiO_2_/EG)
$\mu_{nl}$	$\frac{\mu_{l}}{{\left(1-\varphi \right)}^{2.5}}$
$\rho_{nl}$	$\left(1-\varphi \right)\rho_{l}+\varphi \rho_{s}$
$\rho C_{nl}$	$\left(1-\varphi \right){\left(\rho C_{p}\right)}_{l}+\varphi {\left(\rho C_{p}\right)}_{s}$
$\alpha_{nl}$	$\frac{k_{nl}}{{\left(\rho C_{p}\right)}_{nl}}$
$\frac{\sigma_{nl}}{\sigma_{l}}$	$\frac{\sigma_{s}+2\sigma_{l}-2\varphi \left(\sigma_{l}-\sigma_{s}\right)}{\sigma_{s}+2\sigma_{l}+\varphi \left(\sigma_{l}-\sigma_{s}\right)}$
$\frac{k_{nl}}{k_{l}}$	$\frac{k_{s}+\left(n-1\right)k_{l}-\left(n-1\right)\varphi \left(k_{l}-k_{s}\right)}{k_{s}+\left(n-1\right)k_{l}+\varphi_{1}\left(k_{l}-k_{s}\right)}$

The radiative heat flux $q_{r}$ [see (Chamkha and Ben-Nakhi, 2008), (Sreedevi et al., 2020), (Hamad et al., 2022)] is now simplified using the Rosseland approximation for radiation:

$$q_{r}=-\frac{4\sigma_{*}}{3K^{*}}\frac{\partial T^{4}}{\partial y}$$ (6)

The Stefen–Boltzmann constant and the mean absorption coefficient, respectively, are $\sigma_{*}$ and $K^{*}$ . The temperature differences within the flow are assumed to be such that the term $T^{4}$ may be written as a linear function of temperature. As a result, extending $T^{4}$ in a Taylor series around $T_{\infty }$ while ignoring the higher-order variables yields:

$$\eta =\sqrt{\frac{\left(1+m\right)u_{e}}{2\upsilon_{f}x}}y,\;\psi \left(x,y\right)=\sqrt{\frac{2u_{e}\upsilon_{f}x}{1+m}}f\left(\eta \right),\;\theta \left(\eta \right)=\frac{T-T_{\infty }}{T_{w}-T_{\infty }}$$

$$u=\frac{\partial \psi }{\partial y},v=\frac{-\partial \psi }{\partial x}$$ (7)

Using transformation of similarity Eqs 3–5, 7 becomes

$$\begin{array}{l} C_{1}\varepsilon \left(1+\frac{1}{\beta }\right)\frac{\left(m+1\right)}{2}f^{\prime \prime \prime }\left(\eta \right)+\left(\varepsilon^{2}C_{1}\alpha \left(x\right)\left(1+\frac{1}{\beta }\right)+C_{2}M\right)\left(f^{\prime }\left(\eta \right)-1\right)\\ +\frac{\left(m+1\right)}{2}f\left(\eta \right)f^{\prime \prime }\left(\eta \right)-\left(\gamma \left(x\right)+m\right)\left(f\prime^{2}-1\right)=0 \end{array}$$ (8)

$$\frac{1}{\mathrm{Pr}}\left(C_{3}+\frac{4R}{3}\right)\left(m+1\right)\theta^{\prime \prime }\left(\eta \right)+C_{4}\left(m+1\right)f\left(\eta \right)\theta^{\prime }\left(\eta \right)+C_{5}Ec\left(1+\frac{1}{\beta }\right)f\prime \prime {\left(\eta \right)}^{2}=0$$ (9)

with boundary conditions,

$$f\left(0\right)=0,\;f^{\prime }\left(0\right)=0,\;\theta \left(0\right)=1\;at\;\eta =0,$$

$$f^{\prime }\left(\eta \right)\to 1,\;\theta \left(\infty \right)\to 0\;as\;\eta \to \infty$$ (10)

Initially,

$$C_{1}=\frac{1}{{\left(1-\varphi_{1}\right)}^{2.5}{\left(1-\varphi_{2}\right)}^{2.5}}\left[\left(1-\varphi_{2}\right)\left(\left(1-\varphi_{1}\right)+\varphi_{1}\frac{\rho_{s_{1}}}{\rho l}\right)+\varphi_{2}\frac{\rho_{s_{2}}}{\rho_{l}}\right]$$ (11)

$$C_{2}=\frac{\sigma_{hnl}}{\sigma_{l}}\frac{\rho_{l}}{\rho_{hnl}}$$ (12)

$$C_{3}=\frac{k_{hnl}}{k_{l}}$$ (13)

$$C_{4}=\left[\left(1-\varphi_{2}\right)\left(1-\varphi_{1}+\varphi_{1}\frac{{\left(\rho C_{p}\right)}_{s_{1}}}{\left(\rho C_{p}\right)l}\right)+\varphi_{2}\frac{{\left(\rho C_{p}\right)}_{s_{2}}}{{\left(\rho C_{p}\right)}_{l}}\right]$$ (14)

$$C_{5}=\frac{1}{{\left(1-\varphi_{1}\right)}^{2.5}{\left(1-\varphi_{2}\right)}^{2.5}}$$ (15)

In Eqs 8–10 the superscripts denote differentiation with respect to η . Here $\alpha \left(x\right)=\frac{\upsilon_{lx}}{u_{\infty }K}$ is the first order porous resistance parameter, γ(x)=Fx is the second order porous resistance parameter, $M=\frac{\sigma_{l}B_{0}^{2}x}{\rho_{l}u_{e}}$ is the magnetic field parameter, $\mathrm{Pr}=\frac{\mu_{l}Cp_{l}}{K_{l}}$ is the Prandtl number, $Ec=\frac{u_{\infty }^{2}}{Cp_{l}\left(T_{w}-T_{\infty }\right)}$ is the Eckert number and $R=\frac{4\sigma^{*}T_{\infty }^{3}}{k^{*}k_{l}}$ is the radiation parameter.

Physical quantities skin friction coefficient $C_{f}$ which have now been described as

$$C_{f}=\frac{\tau_{w}}{\rho_{l}u_{\infty }^{2}},\;\text{where}\;\tau_{w}=\mu_{hnl}\left(1+\frac{1}{\beta }\right){u_{y}|}_{y=0}$$ (16)

Using transformation of similarity Eqs 6, 10 becomes

$${Re}_{x}^{1/2}C_{f}=\left(1+\frac{1}{\beta }\right)\sqrt{\frac{\left(m+1\right)}{2}}\frac{f^{\prime \prime }\left(0\right)}{{\left(1-\varphi_{1}\right)}^{2.5}{\left(1-\varphi_{2}\right)}^{2.5}}$$ (17)

and local Nusselt number Nu is described as

$$Nu=\frac{xq_{w}}{K_{l}\left(T_{w}-T_{\infty }\right)},\;where\;q_{w}={-k_{hnl}T_{y}|}_{y=0}$$ (18)

Using Eq 6, Eq 12 becomes

$${Re}^{-1/2}Nu=\frac{-k_{hnl}}{k_{l}}\sqrt{\frac{\left(m+1\right)}{2}}\theta^{\prime }\left(0\right)$$ (19)

## 3 Numerical simulation methodology

The numerical results for cupric oxide—titania/ethylene glycol and titania/ethylene glycol are obtained using MATLAB’s bvp4c function. The work findings are presented graphically, with a focus on the model’s mathematical key components and their effects on velocity, temperature, and engineering interest quantities. The first step is to make a first-order scheme of ODEs out of the third order ODEs (8) to (10).

f=Λ(1) (20)

$$f^{\prime }=\Lambda \left(2\right)$$ (21)

$$f^{\prime \prime }=\Lambda \left(3\right)$$ (22)

$$\begin{array}{l} f^{\prime \prime \prime }=\Lambda {\left(3\right)}^{\prime }=\left\{\frac{\left(m+1\right)}{2}\Lambda (1)\Lambda (3)-(\varepsilon^{2}C_{1}\alpha (1+\beta^{-1})+C_{2}M)(\Lambda (2)-1)+(\gamma +m){\left(\Lambda (2\right)}^{2}-1)\right\}\\ \;\left(-2(C_{1}\varepsilon (m+1)(1+\beta^{-1}){)}^{-1}\right) \end{array}$$ (23)

$$\theta^{\prime }=\Lambda \left(5\right)$$ (24)

$$\begin{array}{l} \theta^{\prime \prime }=\Lambda \left(6\right)\\ =-\mathrm{Pr}\left(\frac{4R}{3}+C_{3}\right){\left(m+1\right)}^{-1}\left\{C_{4}\left(m+1\right)\Lambda \left(1\right)\Lambda \left(5\right)+C_{5}Ec\left(1+\beta^{-1}\right)\Lambda {\left(3\right)}^{2}\right\} \end{array}$$ (25)

with limiting equations

$$\Lambda_{0}\left(1\right)=0,\;\Lambda_{0}\left(2\right)=0,\;\Lambda_{0}\left(4\right)=0$$ (26)

$$\Lambda_{\infty }\left(2\right)=1,\;\Lambda_{\infty }\left(4\right)=0$$ (27)

The function bvp4c requires a first try at the answer because the tolerance for the current issue is set to 10^–8^. The assumption we made must adhere to the solution’s behaviour and satisfy the boundary conditions (26) and (27). Eqs 19–25 are numerically designed in order to get the original value argument to a conclusion. The bvp4c function in the MATLAB software is used to make both of these simplifications.

## 4 Results and discussion

It should be noted that in the current work, ethylene glycol (C_2_H_6_O_2_) is first mixed with nanosized cupric oxide (CuO) particles and then fixed $\varphi_{1}=0.02$ to create a non-Newtonian nanoliquid. To create the hybrid nanoliquid TiO_2_ - CuO/C_2_H_6_O_2_, titania TiO_2_ nanoparticles $\varphi_{2}=0.02$ are added to the nanoliquid and the thermophysical properties of these nanoparticles are mentioned in Table 1. Table 2 compares the findings of Qawasmeh et al. (2019), Chamkha, (1997), Chamkha and Ben-Nakhi (2008), for a given Prandtl number with $\phi_{1}=\phi_{2}=0,$ *m* = 0, *Ec = 0*, ε=1 , α(x)=0, γ(x)=0 , *R = 0* when β→∞ , for Newtonian liquid. A fairly strong consensus can be seen from this table. The Prandtl number is fixed at 204 due to the base fluid being ethylene glycol. The physical parameter values are fixed as $\phi_{1}=\phi_{2}=0.02,$ *m* = 0.0909, *Ec = 0.01*, ε=0.5 , α(x)=0.02, γ(x)=0.01 , *R = 0.5* and β=0.5 throughout the study unless otherwise indicated in the graph.

**FIGURE 2:**
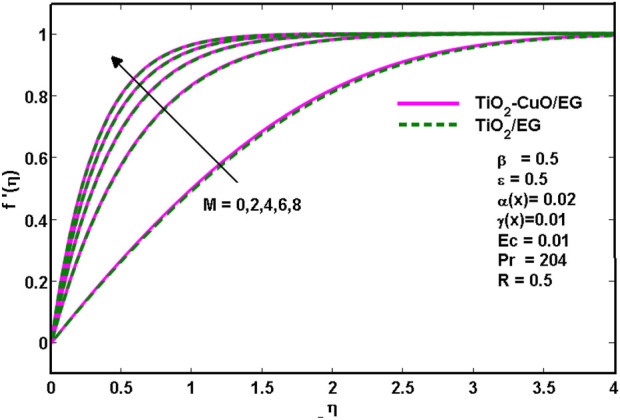
Effects of velocity profiles for various M .

Figure 2 depicts the velocity variation with magnetic parameter *M*. It is evident that as the magnetic parameter increases, the velocity increases as well for the *TiO*_*2*_ */C*_*2*_ *H*_*6*_ *O*_*2*_ and *TiO*_*2*_ *-CuO/C*_*2*_ *H*_*6*_ *O*_*2*_ hybrid nanoliquids. This is due to the Lorentz force, which results in a retarded force on the velocity profile when a magnetic field is present. Moreover, as observed from the figure, the increment is similar for both TiO_2_/*C*_*2*_ *H*_*6*_ *O*_*2*_ and TiO_2_-CuO/C_2_H_6_O_2_.

**FIGURE 3:**
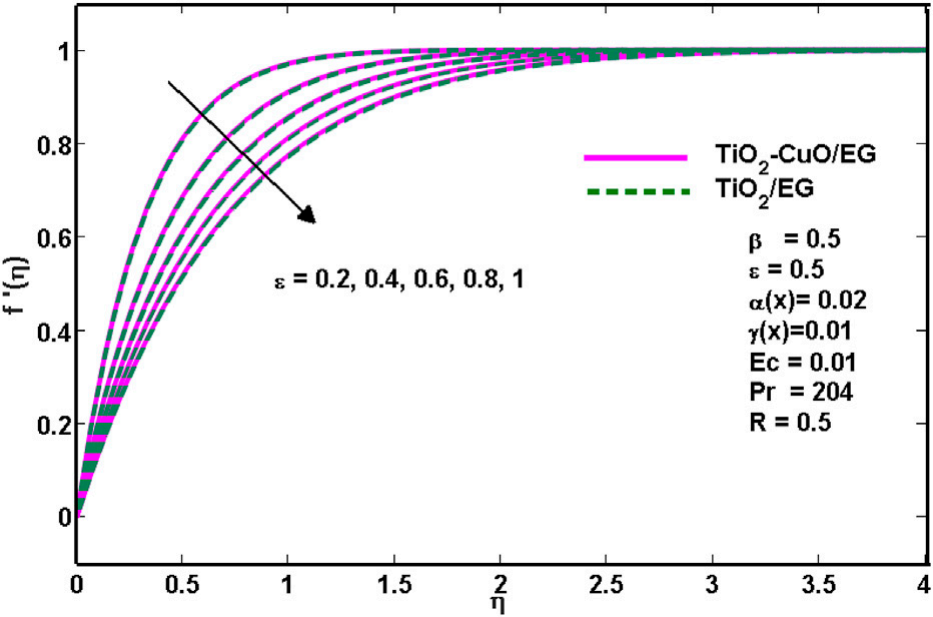
Effects of velocity profiles for various ε .

Figure 3 depicts how the porosity parameter ε affects velocity curves. It is impressive that when ε rises, the nanofluid velocity drops on porous surfaces and its boundary layer widens. The velocity profile gets very close at increased porosity, which is seen in Figure 3 for TiO_2_/C_2_H_6_O_2_ and TiO_2_-CuO/C_2_H_6_O_2_ nanofluids similarly. This pattern is explained by the relatively low permeability and large porous inertia factors.

**FIGURE 4:**
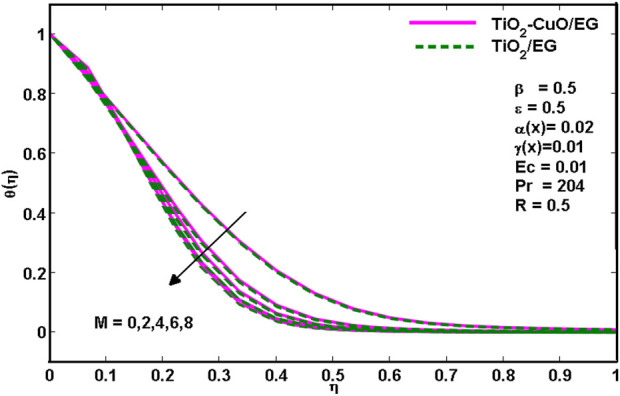
Effects of temperature profiles for various M .

The effect of the magnetic parameter *M* on the thermal distribution is seen in Figure 4. The graph shows that when magnetic parameter values increase, temperature profiles decrease for the *TiO*_*2*_ *-CuO/C*_*2*_ *H*_*6*_ *O*_*2*_ hybrid nanoliquid and the *TiO*_*2*_ */C*_*2*_ *H*_*6*_ *O*_*2*_ nanoliquid. The strong Lorentz force that causes the fluid flow to slow down as a result of the increased development of resistance temperature is caused up to η=0.1 after that lowering the temperature profile.

**FIGURE 5:**
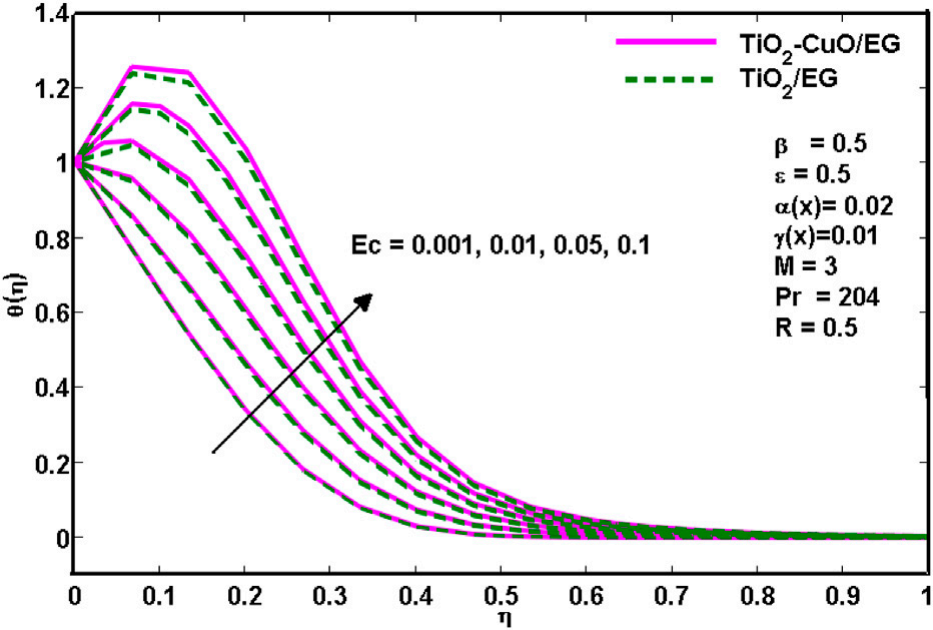
Effects of temperature profiles for various Ec .

Figure 5 shows how the Eckert number *Ec* affects the temperature profile. The Eckert number illustrates the function-versus-viscous fluid tension conversion of external energy into internal energy, which causes the process to be irreversible. It has been established that a rise in the viscous dissipation parameter is accompanied by a rise in temperature. From Figure 5. It is noted that for higher values of the Eckert number, the thermal distribution is a little higher for TiO_2_-CuO/C_2_H_6_O_2_ nanoliquid than for *TiO*_*2*_ */*C_2_H_6_O_2_ nanoliquid.

**FIGURE 6:**
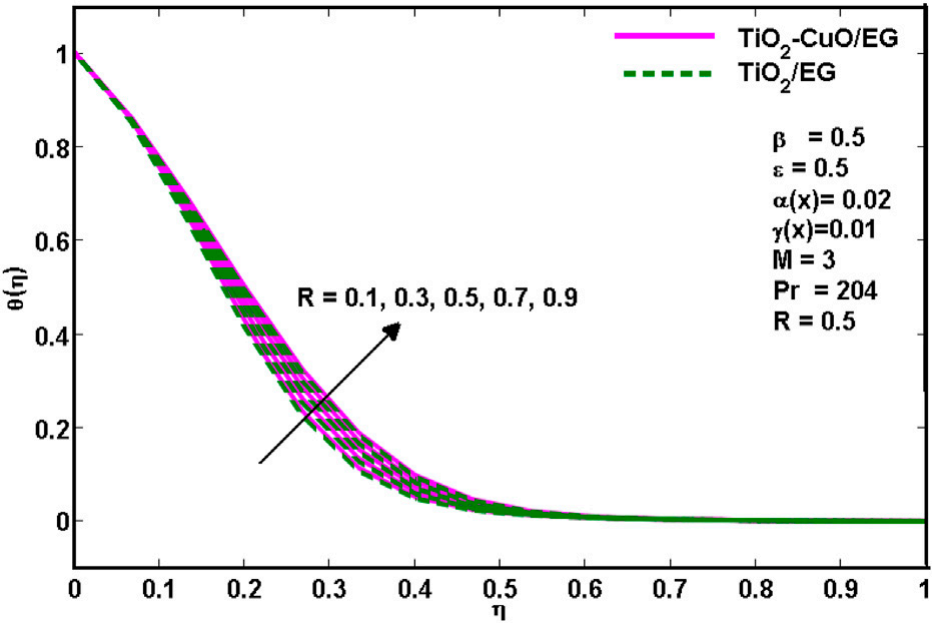
Effects of temperature profiles for various R .

On the other hand, Figure 6. Reveals that the increasing values of radiation parameter (*R*) result in the increasing temperature distribution of the *TiO*_*2*_ *-CuO/*C_2_H_6_O_2_ hybrid nanoliquid and the *TiO*_*2*_ */*C_2_H_6_O_2_ nanoliquid flow region. This is because there would be an increase in the thermal boundary layer thickness with the increase of radiation parameter *R*.

**FIGURE 7:**
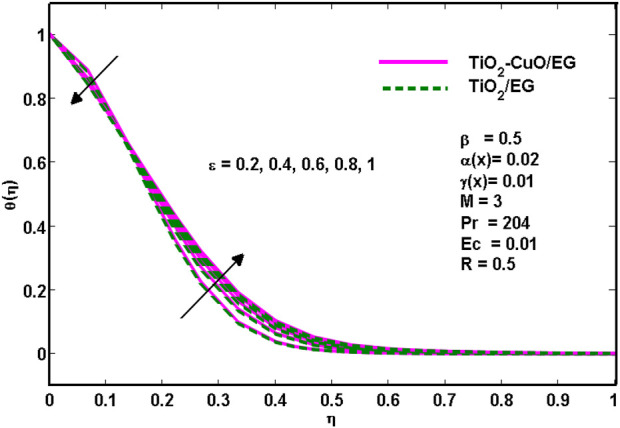
Effects of temperature profiles for various ε .

Figure 7 illustrates how the porosity ε affects the non-dimensional temperature profile. The temperature initially drops as the porosity increases, then it rises. The thickness of the thermal boundary layer also increases with porosity. This behaviour is the same for the mono nanofluid and hybrid nanofluid.

**FIGURE 8:**
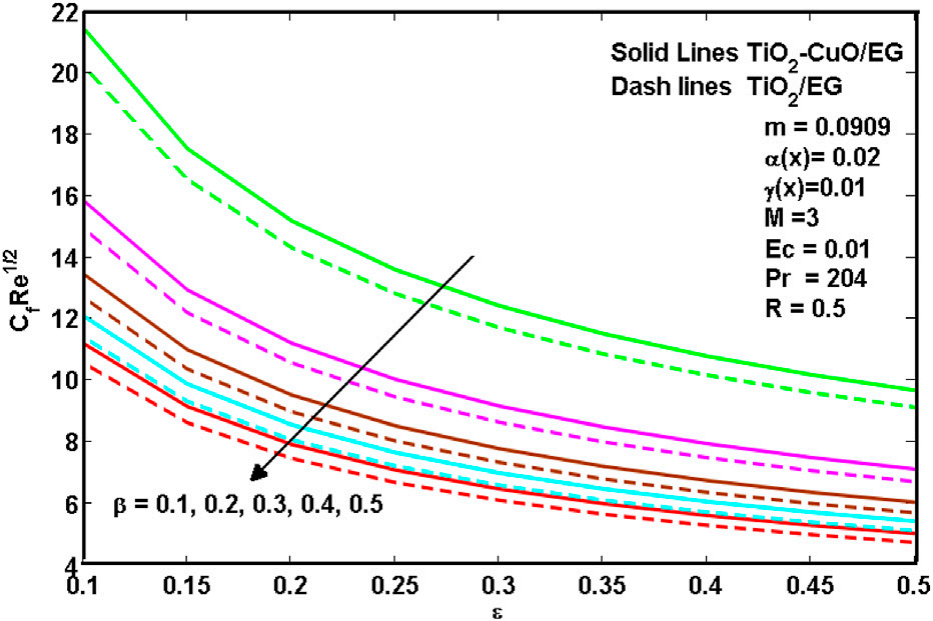
Effects of skin friction for certain ε and β .

**FIGURE 9:**
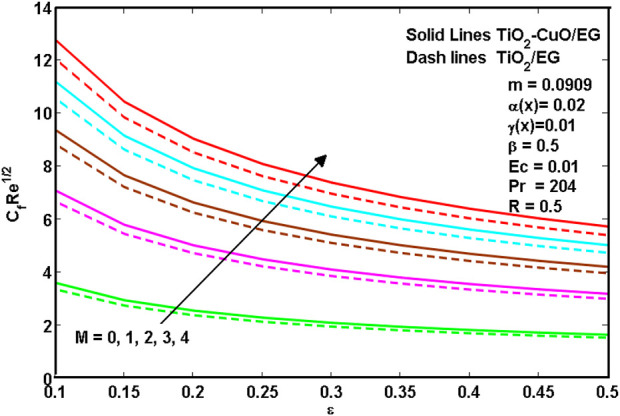
Effects of skin friction for certain ε and M .

**FIGURE 10:**
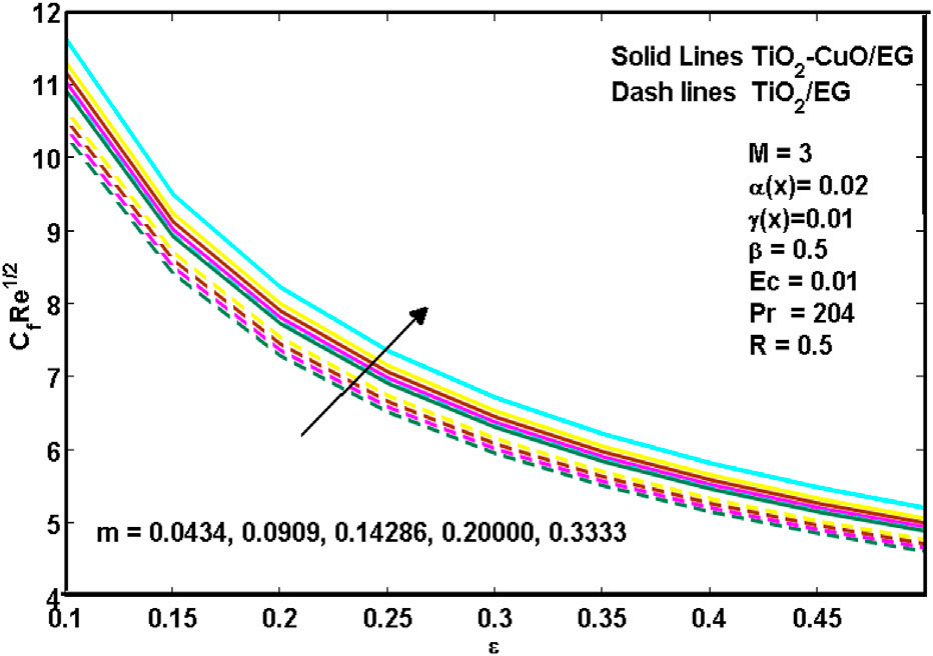
Effects of skin friction for certain ε and m .

Figures 8–10 show hybrid nanoliquid and nanoliquid skin friction past a wedge. It is revealed from Figures 8–10 that the increasing values of porosity ε and Casson parameter β result in a decreasing skin friction coefficient. In the meantime, the increasing values of *m* and *M* result in increasing the skin friction coefficient. Figures 8–10. Illustrate the common trend that the *TiO*_*2*_ *-CuO/*C_2_H_6_O_2_ hybrid nanoliquid has heavier skin friction values than the TiO_2_/C_2_H_6_O_2_ mono nanoliquid. This is due to the fact that the friction made by hybrid nanoparticles *TiO*_*2*_ and *CuO* is greater than mono *TiO*_*2*_ nanoparticles.

**FIGURE 11:**
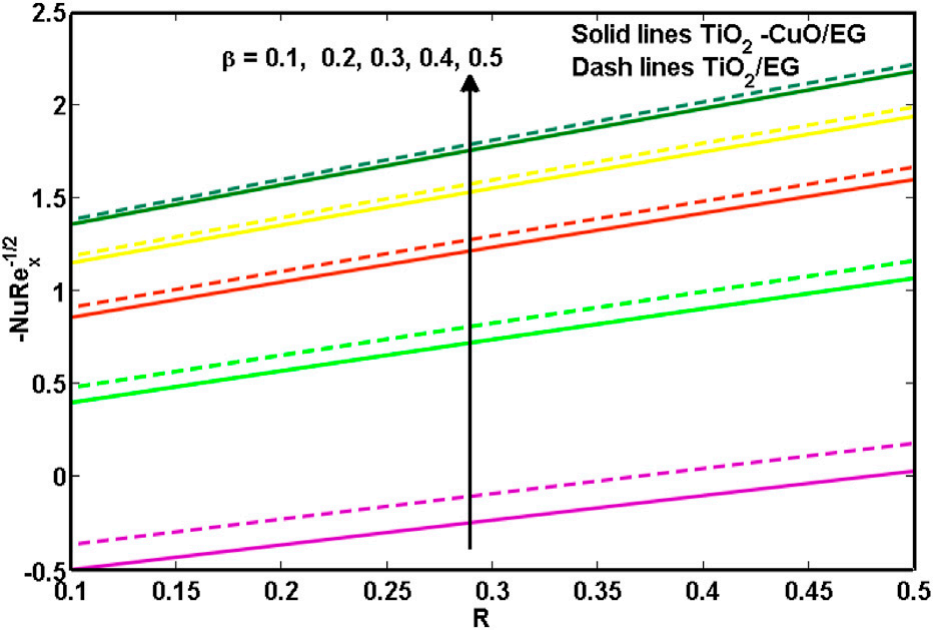
Effects of Nusselt number for certain R and β .

**FIGURE 12:**
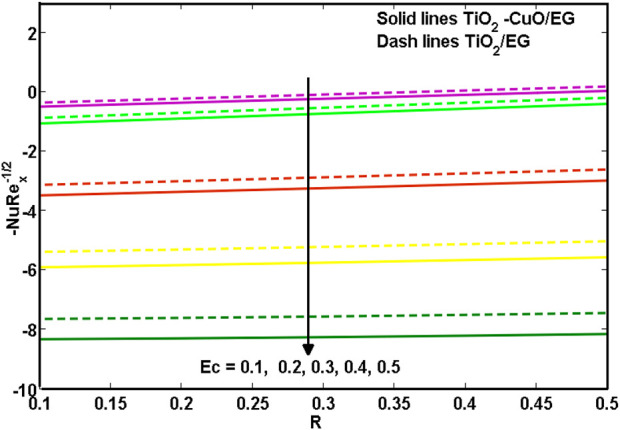
Effects of Nusselt number for certain R and Ec .

Figures 11, 12 illustrate the *TiO*_*2*_ *-CuO/EG* hybrid nanoliquid and the TiO_2_/EG nanoliquid Nusselt number over the wedge for the increment of *R*, β and *Ec*. It is clearly observed that the increasing values of *R*, β and *m* escalate Nusselt number for the *TiO*_*2*_ */EG* nanoliquid than the *TiO*_*2*_ *-CuO/EG* hybrid nanoliquid. Simultaneously, the increasing values of *Ec* drop the Nusselt number. While relating Figures 11, 12, it is revealed that the *TiO*_*2*_ *-CuO/EG* hybrid nanoliquid has a lesser Nusselt number than the TiO_2_/EG mono nanoliquid.

## 5 Conclusion

The momentum and thermal behaviour of viscous dissipative mono and hybrid nanofluids $(TiO_{2}/C_{2}H_{6}O_{2}$ and $TiO_{2}-CuO/C_{2}H_{6}O_{2}$ ) past a wedge embedded in the non-Darcy porous medium with radiation and magnetic field are examined numerically. From the computational results, the following conclusions are given (Table 3).

1) The velocity increases when *M* is increased and decreases when ε is increased for both the nanofluid and hybrid nanofluid.
2) For $TiO_{2}/C_{2}H_{6}O_{2}$ and $TiO_{2}-CuO/C_{2}H_{6}O_{2}$ , temperature rises when ε and *Ec* are enhanced and declines when *M* is raised.
3) An increase in the values of ε and β decreases and the raising values of *M* increases the skin friction respectively.
4) A local Nusselt number is an increasing function for the increasing values of *R* and β.

5) Both the skin friction and Nusselt number increase with the effect of *m.*
6) Greater values of *Ec* lower the Nusselt number for both $TiO_{2}/C_{2}H_{6}O_{2}$ and $TiO_{2}-CuO/C_{2}H_{6}O_{2}$ .
7) While comparing the $TiO_{2}/C_{2}H_{6}O_{2}$ and $TiO_{2}-CuO/C_{2}H_{6}O_{2}$ nanoliquids, the skin friction is a little greater for $TiO_{2}-CuO/C_{2}H_{6}O_{2}$ and the Nusselt number is a little higher for $TiO_{2}/C_{2}H_{6}O_{2}$ nanoliquid.

**TABLE 3:** Amount of $-\theta^{\prime }\left(0\right)$ for certain Prandtl number with $\varphi_{1}=0,\varphi_{2}=0,m=0,Ec=0\varepsilon =1,\alpha \left(x\right)=0,\gamma \left(x\right)=0,R=0$ for Newtonian liquid when β→∞ .

Pr	Current study	Qawasmeh et al. (2019)	Chamkha (1997b)	Alghamdi et al. (2021)
$10^{0}$	0.33226941	0.332054	0.332173	0.332058
$10^{1}$	0.72830834	0.728136	0.72831	0.728148
$10^{2}$	1.57217714	1.571821	1.57218	1.57186
$10^{3}$	3.38788227	3.387073	3.38809	3.38710
$10^{4}$	7.29934030	7.297260	7.30080	7.29742

## Data Availability

The original contributions presented in the study are included in the article/Supplementary Materials, further inquiries can be directed to the corresponding author.

## Nomenclature

### Abbreivations

$C_{b}$: Drag coefficient
$C_{p}$: Specific heat (*J/KgK*)
$u_{e}$: Atmospheric nanofluid velocity
*Ec*: Eckert number
*f*: Fluid
*F*: Forchheimer coefficient
*K*: Permeability
*k*: Thermal conductivity (*W.m* ^*−1*^ *.K* ^*−1*^)
$\mathrm{Pr}=\frac{\mu_{l}C_{P_{l}}}{k_{l}}$: Prandtl number
*T*: Temperature (*K*)
$M=\frac{\sigma_{f}B_{0}^{2}x}{\rho_{f}u_{e}}$: Magnetic field parameter
$R=\frac{4\sigma^{*}T_{\infty }^{3}}{k^{*}k_{l}}$: Radiation Parameter
u,v: x,y velocity components (*m.s* ^*−1*^)

### Greek symbols

α: Thermal diffusivity (*m* ^*2*^ *.s* ^*−1*^)
$\alpha \left(x\right)=\frac{\nu_{l}x}{u_{e}K}$: First order porous resistance parameter
β: Casson liquid parameter
γ(x)=Fx: Second order porous resistance parameter
ε: Porosity
η: Similarity variable
μ: Dynamic viscosity (*N.s.m* ^*−2*^)
ν: Kinematic viscosity (*m* ^*2*^ *.s* ^*−1*^)
ρ: Density (*Kg.m* ^*−3*^)
ψ: Stream function (m^2^.s^−1^)
ϕ: Nanoparticle volume fraction

### Subscripts

hnl: Hybrid nanoliquid
l: Liquid
nl: Nanoliquid
*S*_*1*_: Hard nanoparticle of I nanoparticle of hybrid nanoliquid
*S*_*2*_: Hard nanoparticle of II nanoparticle of hybrid nanoliquid
